# Role of Direct Sexual Contact in Human Transmission of Monkeypox Virus, Italy

**DOI:** 10.3201/eid3009.240075

**Published:** 2024-09

**Authors:** Giuseppe Sberna, Gabriella Rozera, Claudia Minosse, Licia Bordi, Valentina Mazzotta, Alessandra D’Abramo, Enrico Girardi, Andrea Antinori, Fabrizio Maggi, Eleonora Lalle

**Affiliations:** National Institute for Infectious Diseases, Lazzaro Spallanzani, Rome, Italy

**Keywords:** mpox, monkeypox virus, MPXV, viruses, zoonoses, sexual contact, semen, G2R-mRNA, viral load, Italy

## Abstract

The 2022 global mpox outbreak was driven by human-to-human transmission, but modes of transmission by sexual relationship versus sexual contact remain unclear. We evaluated sexual transmission of mpox by using monkeypox virus (MPXV) G2R-mRNA as a marker of ongoing viral replication through in vitro experiments. We analyzed clinical samples of 15 MPXV-positive patients in Italy from different biological regions by using the setup method. The presence of MPXV DNA, MPXV G2R-mRNA, or both in all analyzed lesion swab samples, independent of viral load, confirmed a higher infectivity risk from skin lesions. Positivity for MPXV G2R-mRNA in nasopharyngeal swabs was associated with high MPXV load, whereas positive results for MPXV G2R-mRNA were obtained only in the 2 semen samples with the lowest MPXV loads. Our results suggest that close or skin-to-skin contact during sexual intercourse is the main route of sexual transmission and that semen is a minor driver of infection, regardless of MPXV load.

Monkeypox virus (MPXV) is the etiologic agent of zoonotic mpox disease. Although the virus was first discovered in colonies of monkeys kept for research in 1958, MPXV is mainly transmitted to humans through physical contact with wild infected animals (i.e., squirrels, rats, and mice), with contaminated materials, or with an infectious person (*1*). The first human case of mpox was recorded in 1970 in the Democratic Republic of the Congo; the virus is endemic in central and west Africa, where outbreaks are regularly reported (*1*). 

Before 2022, sporadic mpox cases had been described outside Africa, mainly linked to travel. However, during May–June 2022, the emergence and rapid spread of mpox in >50 countries where the disease was not endemic, led the World Health Organization (WHO) to declare the mpox outbreak a public health emergency of international concern (*2*). The outbreak, caused mostly by the clade IIb variant of the virus, was driven by human-to-human transmission via close contact with infected persons; most cases were described among men who had sex with men. In several studies, viral DNA has been identified and isolated in the semen of infected persons for weeks after they acquired the infection, supporting the hypothesis of sexual transmission (*2*–*4*). Viral DNA was also detected in biologic samples such as saliva, nasopharyngeal swabs (NPS), blood, and urine, thus not always implying the infectivity of the biologic sample (*5*). Now, new in vivo and in vitro models able to mimic aspects of viral biology, such as infectivity, can be developed (*6*). 

Cell culture is considered the standard for virus isolation. Nevertheless, several factors, such as suboptimal sensitivity, eventual long storage of the samples, or the presence of antibodies against the virus in the clinical samples, can contribute to the failure of this procedure, resulting in the inability to establish real viability and infectivity. To improve knowledge about the route of transmission of this infection, finding an alternative method able to overcome problems associated with viral isolation and verify the infective capacity of MPXV in different biological regions is essential.

Considering that MPXV replication strategy is based on a cascade of 3 gene classes (early, intermediate, and late) (*7*,*8*), in this study, we explored the possibility of using an early transcript as a marker of ongoing viral replication. G2R is a crucial gene transcribed during the early phase of infection and can interact with the viral RNA polymerase during the intermediate and late phases of viral replication, thus affecting the fidelity of the transcription process (*8*). Therefore, we selected G2R as a surrogate marker of ongoing replication, because the presence of the G2R-mRNA transcript discriminates between actively replicating and nonreplicating samples, providing an indirect indication of the possible transmission mode.

We conducted a preliminary experiment using Vero E6 cells infected in vitro with MPXV to assess the effectiveness of the developed method for detecting and measuring G2R-mRNA levels. We then analyzed clinical specimens that tested positive for MPXV DNA.

## Methods

### Ethics Statement

This study was conducted in accordance with the Declaration of Helsinki and with protocol code no. 40z, Register of Non-Covid Trials 2022. The study was approved by the Ethical Committee of the Lazzaro Spallanzani Institute MpoxCohort protocol “Studio di coorte osservazionale monocentrica su soggetti che afferiscono per sospetto clinico o epidemiologico di malattia del vaiolo delle scimmie (mpox).”

### In Vitro Experiments

We maintained Vero E6 cells in modified eagle medium supplemented with 10% heat-inactivated fetal calf serum (FCS) at 37°C in a humidified atmosphere of 5% CO_2_ and exposed to MPXV isolate hMpxv/Italy/un-INMI-Pt2/2022, clade/lineage IIb B.1 (GISAID accession no. EPI_ISL_13251120 [https://www.gisaid.org]; GenBank accession no. ON745215.1) for 1 hour and 30 minutes at 37°C at a multiplicity of infection of 0.01. At the end of the adsorption period, we washed and incubated cells at 37°C; at 30 minutes and 1, 2, 3, 4, 6, 24, and 48 hours postinfection (hpi), we harvested, inactivated, and tested cells and supernatants for MPXV DNA and G2R-mRNA presence by digital droplet PCR (ddPCR).

### Clinical Samples

During May–September 2022, a total of 29 samples (7 nasopharyngeal swab, 10 skin lesion swab, 8 semen, and 4 urine samples) were collected for diagnostic purposes from 15 patients admitted to the National Institute for Infectious Diseases (INMI) Lazzaro Spallanzani in Rome, Italy. Patients had a positive diagnosis of mpox within 7 days of symptom onset. We retrospectively analyzed those samples.

### MPXV DNA and G2R-mRNA Quantification

To establish the presence of viral DNA, we first analyzed samples from different anatomic sites of patients with an mpox diagnosis by a commercial MPXV real-time PCR kit (Jiangsu BioPerfectus Technologies Co., Ltd., http://www.bioperfectus.com) on the ELITe InGenius instrument (ELITechGroup SAS, https://www.elitechgroup.com), obtaining a semiquantitative measure by cycle threshold (Ct) value. We further analyzed biological samples that tested positive for MPXV DNA by real-time PCR and cell supernatants from the in vitro experiments by using ddPCR to obtain quantitative results expressed as copies per milliliter. In brief, we extracted 140 μL of supernatant from infected cells and biologic samples by using the QIAamp Viral DNA Mini Kit (QIAGEN, https://www.qiagen.com) according to the manufacturer’s instructions. We quantified MPXV DNA by using the QX200 AutoDG Digital Droplet PCR system (Bio-Rad Laboratories, https://www.bio-rad.com), as previously described (*9*).

To perform G2R-mRNA quantification, we extracted total RNA by using the RNeasy Mini Kit (QIAGEN) according to the manufacturer’s instructions. To selectively degrade any traces of DNA, we treated RNA extracted with DNase (TURBO DNase Kit; Thermo Fisher Scientific, https://www.thermofisher.com). We then reverse transcribed 10 μL of RNA per sample according to the instructions of the SuperScript IV First-Strand cDNA Synthesis reaction kit (Thermo Fisher) by using 50 μmol OLigo d(T)20 as primers to select mRNA. We performed quantification by using the Bio-Rad QX200 AutoDG ddPCR system, targeting the early gene, G2R-mRNA (*10*). To confirm the absence of MPXV DNA fragments, all RNA-extracted samples underwent MPXV DNA PCR after treatment with DNase.

### Statistical Analysis

We performed linear regression analysis by using GraphPad Prism version 9 (https://www.graphpad.com). We expressed results as correlation coefficients (r).

## Results

### In Vitro Cell Culture Experiments

**Figure 1:**
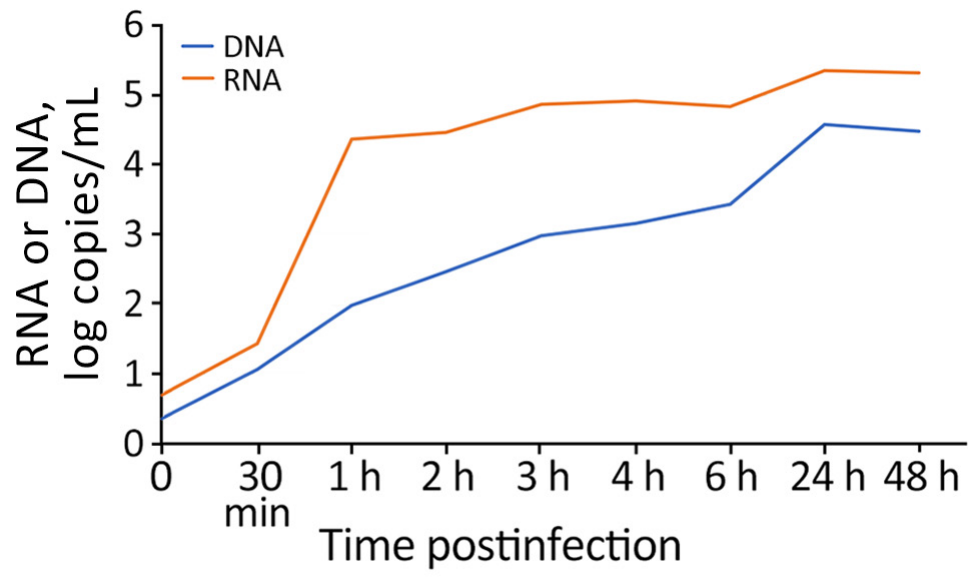
In vitro kinetics of MPXV infection in Vero E6 cell line in study of role of direct sexual contact in human transmission of MPXV, Italy. Cell-associated G2R-mRNA, a surrogate marker of ongoing replication, was detected at low levels until 30 minutes postinfection, when it started to increase, showing a peak at 1 hour postinfection. After 1 hour, a slight increase was observed until 24 hours; levels remained stable thereafter. MPXV DNA levels released in the supernatant steadily increased at each time point, peaking at 24 hours postinfection. MPXV, monkeypox virus.

To establish if G2R-mRNA could be considered a surrogate marker of ongoing MPXV replication, we tested for its presence in vitro in infected Vero E6 cells. Cell-associated G2R-mRNA was detected at low levels until 30 minutes postinfection, when it started to increase, showing a peak at 1 hpi (Figure 1). This result was expected because the G2R gene is early transcribed during viral infection. After 1 hpi, we observed a slight increase in cell-associated G2R-mRNAs throughout the infection until 24 hpi; levels remained stable thereafter. All RNA samples treated with DNase were negative for MPXV DNA, confirming the absence of MPXV DNA fragments and the strength of the setup method (data not shown).

MPXV DNA levels released in the supernatant steadily increased at each timepoint, peaking at 24 hpi. We observed substantially lower levels of DNA released in the supernatants for mRNA cell-associated DNA, starting at 30 minutes postinfection and continuing throughout the observation period.

### MPXV DNA and G2R-mRNA in Clinical Specimens

**Figure 2:**
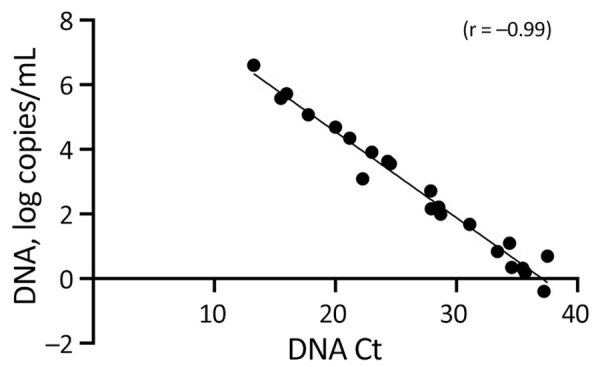
Linear regression analysis of Ct values and MPXV DNA levels in clinical specimens from study of role of direct sexual contact in human transmission of MPXV, Italy. Results show highly negative correlation (r = −0.99) between Ct values and the amount of MPXV DNA. Ct, cycle threshold; ddPCR, digital droplet PCR; MPXV, monkeypox virus.

MPXV DNA and G2R-mRNA were present in NPS, skin lesion swabs, urine, and semen samples (Table). All samples were positive for MPXV DNA by diagnostic real-time PCR (mean Ct 27.3, range 13.3–37.7), except for all urine and 2 semen samples that showed negative results (Ct >45.0). We performed absolute quantification of positive MPXV DNA samples by ddPCR, and linear regression analysis found a highly negative correlation (r = −0.99) between the Ct values and the amount of MPXV DNA (Figure 2).

**Table:** Molecular results of analyzed samples in study of role of direct sexual contact in human transmission of MPXV, Italy*

Sample type	Patient no.	MPXV DNA Ct	MPXV DNA ddPCR, copies/μL	G2R-mRNA ddPCR, copies/μL
Semen	1	24.5	3,576	ND
	2	34.5	2.2	2.4
	4	ND	NT	NT
	5	ND	NT	NT
	6	27.9	145.2	ND
	8	28.7	98.8	ND
	11	34.3	12.4	6.0
	12	37.7	ND	ND
Skin lesion swab				
Penis	1	22.2	1,216	7.2
Cutaneous	2	13.2	4,000,000	504
Penis	3	24.3	4,296	3.6
Scapula	5	35.4	2.1	2.8
Anal	9	35.6	1,552	4.8
Cutaneous	10	15.5	380,000	608
Anal	10	20.0	49,060	79.6
Pubis	13	15.9	521,000	16.4
Neck	13	17.7	117,600	10.8
Back	15	23.0	8,188	4.4
Nasopharyngeal swab	1	21.2	22,080	13.2
	2	31.1	48	1.2
	4	27.8	520	1.6
	5	37.5	4.9	ND
	6	28.5	164.4	10
	7	37.2	1.0	ND
	14	33.3	6.9	ND
Urine	1	ND	NT	NT
	2	ND	NT	NT
	5	ND	NT	NT
	6	ND	NT	NT

*Ct, cycle threshold; ddPCR, digital droplet PCR; MPXV, monkeypox virus; ND, not detected; NT, not tested.

G2R-mRNA was detectable in all skin lesions (r = 0.71) and 4 of 7 NPS samples (r = 0.64), showing a good correlation with MPXV DNA in both matrices. Of note, we observed low positivity for G2R-mRNA presence in only 2 of 8 semen samples; therefore, we could not correlate those results with DNA levels. Nevertheless, we emphasize that semen samples with the highest DNA copy numbers tested negative for G2R-mRNA, which is different from what we observed in other matrices (Table).

## Discussion

During January 1, 2022–February 2024, more than 94,000 laboratory-confirmed cases of mpox, including 181 deaths, were reported to WHO from 117 member states across all 6 WHO regions (*11*). Cases and sustained chains of transmission have been reported concurrently in nonendemic and endemic countries in widely disparate geographic areas and have involved mainly, but not exclusively, men who have sex with men. Although close physical contact with lesions on the skin or mucosal surfaces of mpox-symptomatic persons represents the main factor for human-to-human transmission in this outbreak (*12*), recent studies suggest that sexual activity could represent an important route of disease transmission (*2*,*13*). Lesions on the genitalia, perianal, and inguinal areas of infected persons that tested positive for MPXV DNA (*4*,*14*), as well as the high prevalence of positivity in semen samples from mpox cases (*9*,*15*), have been considered further evidence supporting the sexual transmission route. A correlation between the amount of viral load and infectious virus titer has already been described for lesion and NPS swab samples (*16*); on the contrary, difficulties in viral isolation were found for semen samples, even when the viral load was high (*17*). That evidence opens the issue concerning the difference between sexual and sexual contact transmissions. To overcome problems associated with viral isolation and to verify the real infectious capacity of MPXV in different biological regions, we first evaluated the possibility of using MPXV G2R-mRNA as a marker of ongoing viral replication through in vitro experiments. The presence of high levels of G2R-mRNA during the early phase of infection (within 1 hour), associated with low levels of MPXV DNA, confirms that this method reflects the replication strategy of MPXV, because G2R is an early gene. Therefore, we applied the same method in vivo to analyze clinical samples from different biological regions of MPXV-positive patients.

Our results showed the presence of either MPXV DNA or MPXV G2R-mRNA in all analyzed lesion swab samples, independent of MPXV DNA load, thus confirming a higher infectivity risk from skin lesions. As far as NPS samples are concerned, the presence of MPXV G2R-mRNA was associated with a high MPXV DNA load, indicating higher infectivity in NPS samples with low Ct values (*16*). When analyzing semen samples, we obtained positive results for MPXV G2R-mRNA in only the 2 samples with the lowest MPXV DNA level, suggesting that this biologic fluid could represent a minor route in the context of sexual transmission, regardless of viral load. A possible explanation for the other 6 semen samples showing high MPXV DNA levels in the absence of viral replication (i.e., negative MPXV G2R-mRNA) could be a passive diffusion from skin lesions on the genitals or hands (*18*). In fact, when analyzing data coming from patient 1, we observed a lesion on the penis with a high viral load (Ct 22.2) and a clear positivity for the presence of G2R-mRNA, indicating active replication in that site. Nevertheless, semen from the same patient showed a high viral load (Ct 24.5), in the absence of active replication (negative G2R-mRNA), with negative urine samples, suggesting possible contamination of semen from a lesion on the penis.

In conclusion, the use of MPXV G2R-mRNA as a marker of replication enables discrimination between infected and contaminated samples, overcoming the problems related to viral isolation and providing an explanation for the difficulty encountered in isolating the virus from semen samples even with a high viral load (*17*). Despite the limited number of tested samples, our data support the evidence that sexual contact is the main route of sexual transmission, whereas semen samples can represent a minor driver of infection, independent of MPXV DNA load. This evidence is crucial to enable development of proper interventions and to provide valuable support for decision-making regarding protective measures for mpox patients and their close contacts.
